# Estimation of Surface Roughness on Milled Surface Using Capacitance Sensor Based Micro Gantry System through Single-Shot Approach

**DOI:** 10.3390/mi13101746

**Published:** 2022-10-15

**Authors:** Rajendran Mathiyazhagan, SenthamaraiKannan SampathKumar, Palanisamy Karthikeyan

**Affiliations:** 1 Department of Production Technology, Madras Institute of Technology Campus, Anna University, Chennai 600044, Tamil Nadu, India; 2 Department of Mechanical Engineering, College of Engineering Campus, Anna University, Chennai 600025, Tamil Nadu, India

**Keywords:** roughness estimation, capacitance technique, FEA analysis, micro gantry

## Abstract

The profile generation is highly complex for roughness measurement using a capacitive sensor because of the small peak-to-peak width of the machined surface and the close proximity of the sensor setting with the machining setup which has the chance of damaging the sensor. Considering these shortcomings, a higher sensor sensing diameter with an appropriate resolution has been selected for a single-shot approach. An automated micro gantry XYZ system is integrated with a capacitive sensor to precisely target, move, and measure the roughness. For investigation, a vertical milled surface with a wide roughness range has been prepared. A Stylus profilometer has been used to measure the roughness (*R_a_*) of the specimens for comparison. An experiment has been conducted on the above system with a 5.6 mm capacitance sensor, and an estimation model using regression has been obtained using sensor data to estimate *R_a_*. In conclusion, the single-shot approach with a 5.6 mm sensing diameter sensor, the proposed micro gantry system, and the estimation model performs better in instantaneous noncontact measurement in the range of 0.3 µm to 2.9 µm roughness estimation. The influence of tilt and waviness has also been discussed using FEA analysis.

## 1. Introduction

The wear, friction, lubrication, coating, and appearance of a machined object are all influenced by the surface profile. The surface parameter will also aid production engineers in measuring and controlling the surface finish of produced components [1,2,3,4,5,6]. If we think about control in a manufacturing process, then the measurement should be online. To assess the functional property of the machined surface, the predominantly used surface finish parameter is arithmetic average height (*R_a_*) [7]. Methods to measure surface roughness parameters are broadly classified into contact and noncontact types. For offline measurement, well-established contact-type method such as the stylus profilometer method were used [8], and noncontact methods using optical techniques such as vertical scanning interferometry (VSI) and the confocal method were preferred for profile measurement as well as areal surface roughness measurement [9]. However, the stylus method has limitations such as the need to change the tip of the stylus for different roughness ranges, as contact over the surface will cause damage to the machined specimen and the stylus itself, and for single probing optical methods such as VSI and confocal methods, standard laboratory conditions for measurement are required, which limits the ability to use these methods in online conditions. For the past three decades, researchers have worked on area integration techniques such as pneumatic techniques [10,11] and inductive sensor [12]; optical, laser scanning, and machine vision techniques have been used. Pneumatic and inductive methods have disadvantages such as orientation and material dependence, respectively. The optical method uses five phenomena such as relative intensity or reflectance in the specular direction, the total intensity of the scattered light [13], the diffuseness of the angular scattering pattern, the speckle contrast, and the polarization, which are dependent on the surface roughness and have served as the foundation for potential surface measuring instruments [14,15,16]. A machine vision technique has been employed in surface roughness prediction. The statistical characteristics were generated from the grey-level intensity histogram of the machined surface image and associated with the stylus-determined average surface roughness (*R_a_*) value [17]. Researchers have also investigated the use of texture parameters and the statistical color distribution matrix of the obtained image from the machined surface [18,19,20]. The optical and machine vision approaches, on the other hand, have the limitations such as directionality, reflectivity of the surface, lighting conditions, and positioning of the setup for measurement. Different machining processes may have an impact on the surface pattern [21,22]. To overcome the above-mentioned difficulties, this work was planned to investigate the possibility of using a capacitance sensor to estimate the surface roughness of a vertically milled specimen.

The first attempt was made by Sherwood et al. [23] to obtain a theoretical relation to assess the surface finish using the area method capacitance technique. Garbini et al. used a fringe field capacitance sensor (FFC) for surface finish measurement in the turning process [24,25]. Changes in capacitance are caused by varying distances while moving a probe along the surface. This is basically a contact-type technique, and it is affected by the burrs present on the workpiece. Saji Varghese et al. used pulse jet capacitance sensors along with inductive and fiber optic sensors to predict the roughness using a neural network. This work was performed to generalize the prediction system for all materials and processes [12]. However, the making of measurements in the same place by all the sensors and the effect of distilled water in the pulse jet capacitance sensor on inductive and optical measurement were not addressed, and many samples were required to train the system for generalized roughness measurement. Guadarrama-Santana et al. performed experiments with flat and corrugated electrodes. The change in capacitance for a change in roughness was reported in this work [26]. However, this work was not carried out on the machined surface. Murugarajan et al. performed experiments to model and evaluate a capacitance-sensor-based surface roughness system. The experiment was performed using a 0.5 mm sensing diameter capacitance sensor on ground, milled, and shaped surfaces to generate the profiles of the surfaces [22]. The report shows that the sensor performs better for a ground surface than for milled and shaped specimens. The work concludes that the sensor diameter should be less than the peak-to-peak distance to generate a profile. As a part of the investigation of our previous work, the sensor model with a 0.5 mm sensing diameter was developed in Ansys Maxwell, ANSYS, USA and the capacitance response for ground, milled, and turned surfaces was studied by considering three different stand-off distance (SODs). The SOD of 50 µm had the highest correlation factor for the three machined surface models [27]. However, the experimental work was not performed using this 0.5 mm market-available capacitance sensor because of the requirement for a closer SOD to the workpiece, which makes this sensor selection not suitable for online conditions. The sensor with sensing diameter of 0.5mm is not close enough to the evaluation length of 4 mm mentioned in ISO4288/ISO4287 for *R_a_* measurement around 2µm and may not be an appropriate choice for a higher roughness range. 

Even though researchers have reported the change in capacitance for change in roughness, an attempt to reproduce a profile close to a stylus-generated profile using the FFC method and the parallel plate capacitance method has not been made. Most of the previous work mainly discusses obtaining roughness from a generated profile of a machined surface. However, the pattern of capacitance changes for roughness change in single-shot measurement, the selection of sensor sensing diameter for single-shot measurement, and the selection of an appropriate stand-off distance have been not investigated sufficiently. This single-shot approach has the advantage of VSI and confocal area topography measurement, which use a single probe to capture the area information [28]. These two optical methods give spatial and amplitude information about the surface. However, the capacitance technique in the single-shot approach will give area-integrated information. As mentioned in the ISO4287/4288 standards, to measure various ranges of roughness, the stylus tip needs to be selected accordingly [29]. However, for noncontact methods, which mostly involved optical methods such as vertical scanning interferometry, the confocal microscope method uses 3D standard ISO25178. The 3D standard does not determine the area to be considered for measurement of particular roughness as in 2D roughness parameter measurement. For choosing the capacitance sensor diameter, the evaluation length considered in the 2D surface roughness parameter has been considered. For most applications, the surface roughness *R_a_* has the range of 0.4 µm to 3.2 µm, for which the evaluation length for 2D profile measurement is 4 mm. Considering this as one of the important factors, the sensor diameter for the experiment has been chosen as 5.6 mm. The work was further extended to determine the better roughness measurement range for a 5.6 mm sensing diameter sensor and also the influence of parameters such as stand-off Distance (SOD), sensor diameter, and FEA analysis for studying changes in capacitance and influence of tilt and waviness in roughness measurement. 

## 2. Materials and Methods

### 2.1. Study on Capacitance Response for Rough Surface 

**Figure 1:**
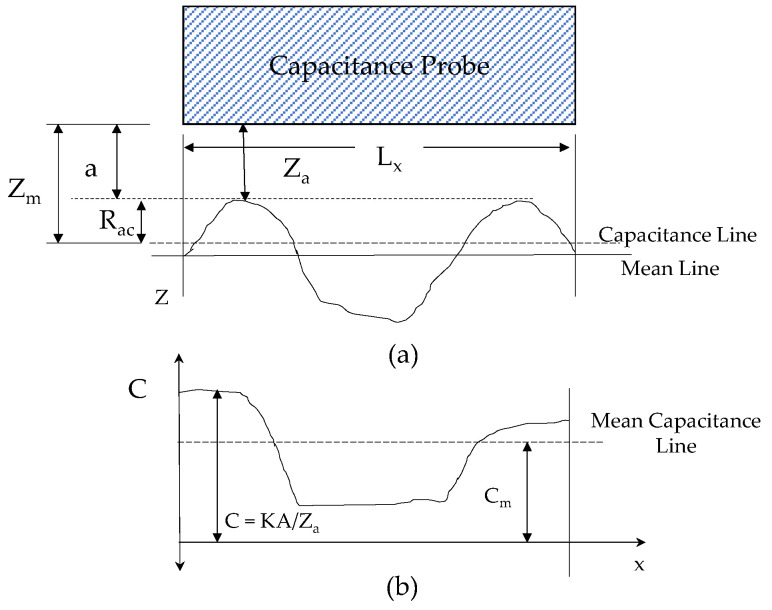
Capacitance sensor principle for surface roughness measurement [23] (**a**) Z-Plan of the Profile (**b**) Corresponding Capacitance Plan for the Profile.

Changes in the dielectric medium, the overlapping area, and the distance between the two electrodes have all been implicated in the capacitance variation observed in the parallel plate capacitance sensor. The surface roughness was determined by employing a capacitance displacement measuring sensor in this study. As indicated in Figure 1a, the plane section of the surface profile indicates the probe placement over the rough surface with the stand-off distance (SOD) of “a”. The SOD indicates the distance between the machined surface peak point and the sensor sensing electrode and Figure 1b shows the corresponding capacitance plan of the profile. The dimensionless equivalent of the average capacitance C*_m_* is *Z_m_*. 

The capacitance (*C*) equation is shown in Equation (1), where *A* is the area of the probe sensing diameter overlapping over the rough surface, dxdy is the small amount of specimen area separated by the distance *Z_a_*, and *K* is the dielectric constant. The capacitance for the probe is given as

(1) $$C=K\iint_{0}^{L_{x}L_{y}}\frac{dxdy}{Z_{a}}=\frac{KA}{Z_{m}}$$

The *Z_m_* is the mean value of the surface for the deviation of points from the center line average (c.l.a). *Z_m_* is the reciprocal of the mean value of 1/*Z_a_*. The relation is given in Equation (2). The capacitance increases when the surface is closer to the electrode. The surface profile function can be written as *Z_a_* = f(x,y); on adding the stand-off distance (SOD), the *Z_a_* equation has been remodified as *Z_a_* = a + f(x,y). When the SOD is zero, *Z_a_* is equal to the profile function.

(2) $$\frac{1}{Z_{m}}=\frac{1}{A}\iint_{0}^{L_{x}L_{y}}dxdy/Z_{a}=\frac{1}{A}\iint_{0}^{L_{x}L_{y}}dxdy/\left(a+f\left(x,y\right)\right)$$

where *A* = *L_x_L_y_*, the overlapped area between the probe and the surface. The capacitance sensor sensing component acts as one electrode, while the machined surface acts as another electrode. *R_ac_* is the roughness measured using the capacitance technique for which Equation (3) gives the relation between mean distance variation due to roughness (*Z_m_*) and stand-off distance (*a*).

(3) $$Z_{m}=R_{ac}+a$$

For a “nominal” spacing between a rough surface and the probe, the observed (mean) capacitance (*C_m_*) is given by Equation (4). Consequently, a simple, generalized link between the capacitance line and other “direct” profile characteristics such as peak-to-valley height and center line average (c.l.a.) height is not possible at this time [22,23]. However, *C_m_* is inversely proportional to *Z_m_*. f(x,y) is the function that represents the roughness profile, waviness, and tilt in the surface, and no solution has been discovered to separate waviness and tilt to determine the change in capacitance purely due to roughness.

(4) $$C_{m}=KA\overset{L_{x}L_{y}}{\underset{0\;}{\iint }}\frac{dxdy}{a+f\left(x,y\right)}$$

As shown in Figure 1, the probe SOD has been fixed from the peak of the machined profile. However, finding the mean line of the surface profile as in the stylus method is not possible. It is clear from the theory that a greater number of peaks moves the *C_m_* and *Z_m_* planes closer to the sensor. For this reason, the *R_ac_* value has to decrease as roughness increases. This has been experimentally verified and also analyzed using FEA analysis.

### 2.2. Proposed Methodology

**Figure 2:**
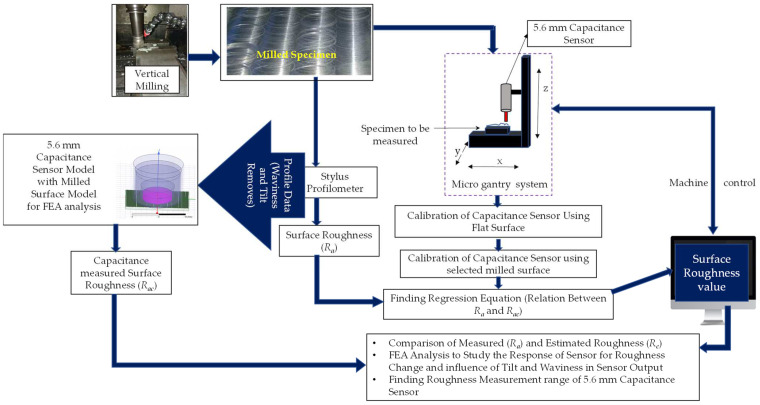
Methodology.

This work proposes the single-shot approach to estimate the roughness of vertical milled specimen. The methodology has been shown in Figure 2. The vertically milled specimens prepared with machining conditions such as different speeds, feed rates, and depths of cut to obtain various roughness using the same cutting tool, as discussed in Section 2.3. An obtained specimen with varying roughness was measured using a stylus profilometer. The predominantly used surface roughness parameter arithmetic average height (*R_a_*) was measured using a stylus profilometer for each specimen in three different parts of the marked region, and the average of the three roughness values was considered as ground truth. The stylus measurement steps and their details are discussed in Section 2.4. The selected capacitance sensor was calibrated against an optical flat surface, and the experiment was repeated in real time with the help of an XYZ table with both the selected vertically milled specimen and the remaining vertically milled specimen by setting the appropriate SOD using a gantry-type XYZ stage. The experimental setup and the experimental steps are discussed in Section 2.6. The capacitance sensor was calibrated with the selected specimen to find a regression model predicting the roughness of the other machined specimen. 

The results and discussion of experimentation with flat and vertically milled specimens using a 5.6 mm capacitance sensor are presented in Section 3, along with a discussion related to roughness estimation on the remaining specimen, selection of the sensor (sensing diameter), FEA simulation to assess the effect of tilt and waviness, and stand-off distance selection. The work is concluded with findings, suggestions, and future work in Section 4.

### 2.3. Specimen Preparation

**Figure 3:**
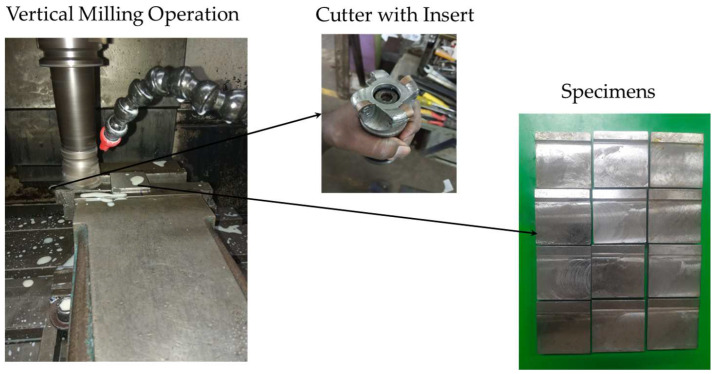
Vertical milling machining setup.

Steel alloy EN31, which has wide application [30,31], was selected as the material. Thirteen specimens were prepared by varying the speed, feed rate, and depth of cut in vertical milling. The R8 ball nose Sandvik-made GC1040 PVD coated grade insert, Sandviken, Sweden, with a 50 mm end mill cutter was used for machining. The milling process parameter was considered under various levels of speed (500, 1000, and 1500 rpm), feed rate (150, 300, and 500 mm/min), and depth of cut (0.5, 1, and 1.5 mm). This machining setup and the machined specimen are shown in Figure 3. The roughness measurement on the machined specimen over the laser-marked place was carried out using a stylus profilometer, which is explained in Section 2.4.

### 2.4. Stylus Measurement Setup

**Figure 4:**
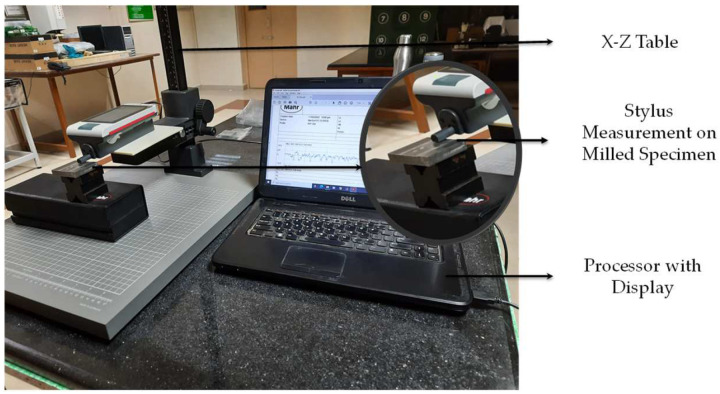
Stylus profilometer.

For measurements, a Mahr PS10(3053) contact-type stylus profilometer (Deutschland, Germany) and a PHT350 probe with a measuring error of 2% and a sensor probing system resolution of 8 nm with a measurement range of 350 µm with a positional speed of 0.5 to 1.0 µm/s were used. The probe is an inductive skidding probe. The setup is shown in Figure 4. The stylus tip, cut-off length (c), and evaluation length (Ls) as specified in ISO4288/16610-21 were considered for assessing the surface roughness of the vertically milled specimen. *R_a_* was measured in a designated specimen region. Nearly 9600 points were sampled across the evaluation length to generate the profile, and the respective peaks and valleys were selected within the cut-off length by software as per standard to compute *R_a_*. The measurement also yields height, spatial, hybrid, and functional characteristics. The height parameter *R_a_* was considered for comparison and regression model development.

### 2.5. Calibration of Capacitance Sensor with Flat Surface 

**Figure 5:**
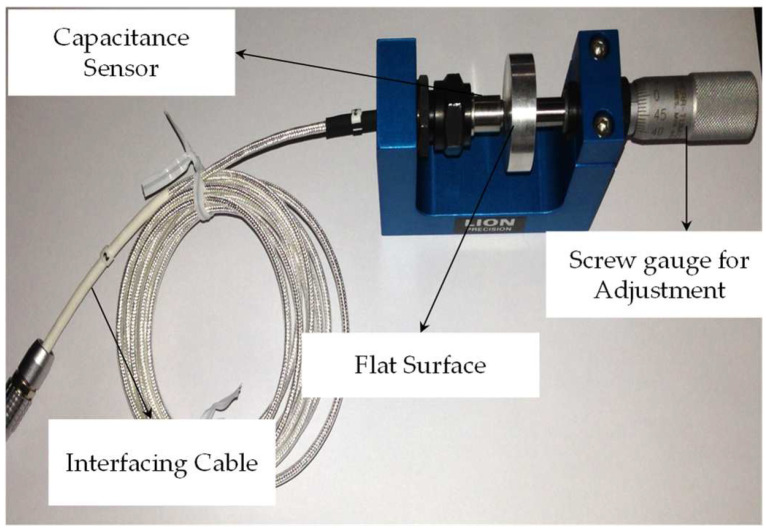
Hardware setup for sensor calibration.

Figure 5 shows the configuration used to calibrate the sensor for a flat surface. Attached to the screw gauge is a fine flat surface of steel. At the opposite end of the fixture, a capacitive sensor is attached. Sensors are able to detect movement on a flat surface. Using the supplier’s calibration chart, we increased the stand-off distance for the C1-A lion precision capacitance displacement sensor from the original minimum of 250 microns to a 2250-micron range with the periodic distance, and the resulting voltage output was used to determine the relation between output voltage (*V*) and distance moved (D). The results are discussed in Section 3.

### 2.6. XYZ Micro Gantry System for Roughness Measurement and Experimental Procedure

**Figure 6:**
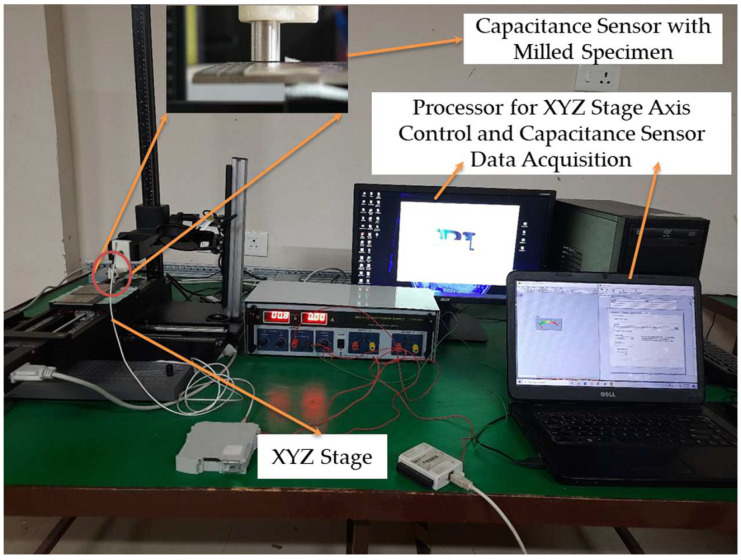
Micromovement gantry setup for roughness measurement using capacitance sensor.

The market-available Lion Precision C1-A capacitance displacement sensor with a 5.6 mm sensing diameter was selected for single-shot measurement. After calibration of the sensor, as discussed in Section 2.4, the sensor was fixed on the measurement setup as shown in Figure 6. The capacitive sensor was fixed using a holder on the Z-axis, which was made using a uPrint SE Plus 3D printer(Minnesota, USA). The specimen was placed on the XYZ stage, which was driven by a PI (Physik Instrumente) controller (Karlsruhe, Germany). The PIMikroMove software version 2.11 was used to control the XYZ stage. Based on the calibration chart and the sensitivity of the capacitance sensor, the appropriate SOD selected was 400 µm for this application. The setting of the SOD for each specimen was carried out by using PIMikroMove software to position Z-axis from the specimen. The capacitive sensor had a driver that sent out a 0–10 V output based on how far away the target plate was from the sensor’s sensing area. The output from the capacitance sensor driver module was linked to the LabVIEW software version 2020 through a USB6009 National Instrument data acquisition card (DAQ) manufactured by NI, Texas, USA. For single-shot measurements, the DAQ was set to 1000 samples per second, the sensor was set to different stand-off distances, and the data were collected for calibration. During experiments with a rough surface, the sensor was fixed at a selected stand-off distance for measurement. The data were saved for further analysis. The XYZ stage and sensor specifications are presented in Table 1.

Procedure followed to Estimate Roughness Using Capacitance Sensor:

Measurement steps are listed as follows for all vertically milled specimens for surface roughness estimation using the capacitance sensor:

- The specimen was prepared using a vertical milling process for varying roughness by varying speed, feed rate, and depth of cut.
- Laser marking was made the specimen with the dimensions of 6 mm × 6 mm for performing the measurement with both the stylus and capacitance sensor on the same spot.
- Measurement using a stylus profilometer was performed within the laser-marked spot.
- The sensor, which had been calibrated using a flat surface, was fixed in the XYZ stage.
- The laser-marked part of the specimen was exactly placed under the calibrated capacitance sensor for the selected stand-off distance (SOD) for measurement.
- The voltage data for the particular spot of the machined surface were recorded using LABVIEW software and the data acquisition card.
- Steps 1 to 6 were repeated for all 13 specimens used. Six of these specimens with a wide roughness range were selected for calibrating the sensor for a wide roughness range.
- For the voltage obtained for the selected 6 specimens, *R_a_* was estimated in two ways.
  - Firstly, the relation between roughness (*R_a_*) and voltage (*V*) was obtained using a regression model. This relation was used to estimate roughness for the remaining specimen.
  - Secondly, the obtained voltage was converted to distance by using Equation (6). The stand-off distance needed to be subtracted from the obtained distance (*Z_m_*) to determine *R_ac_* as shown in Equation (2). Then, the relation between *R_a_* and *R_ac_* was determined using regression.
- The measurement performed using the capacitance sensor for the remaining 7 specimens was substituted in the regression model to estimate the roughness *R_a_*.

The measurements performed and the *R_c_* values estimated are tabulated in Section 3, and a discussion of the results is also presented. 

## 3. Results and Discussions

The results of the experiment performed by following the procedure discussed in Section 2.6 are presented and the observations and findings from the results are discussed in this session. 

### 3.1. Calibration of C1-A Capacitance Sensor using Flat Surface

**Figure 7:**
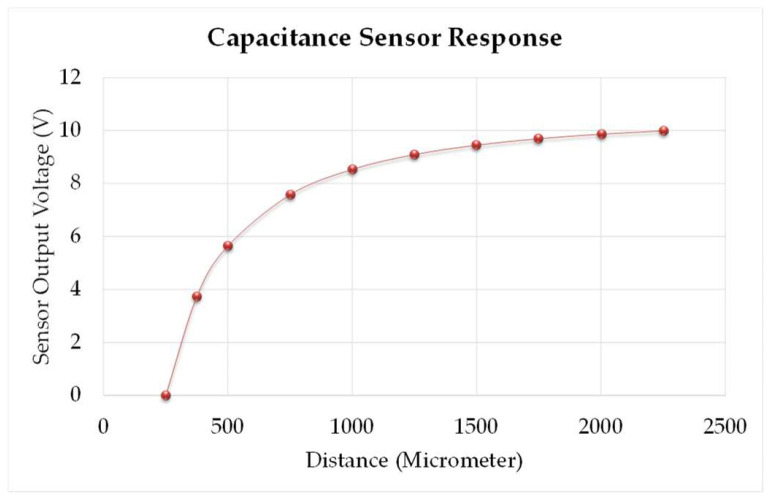
Response of capacitance sensor for different positions of flat surface.

Before using the capacitance sensor for measurement, the sensor calibration has been performed using a calibration stage for different positions as mentioned in the calibration chart by the manufacturer. Table 2 exhibits the voltage (*V*) for the periodic distance (D) using the calibration stage. The surface has been moved periodically from 250 µm to 2250 µm distance. Figure 7 depicts the response, and Equations (5) and (6) show the model equation obtained by plotting the best fit line for the output voltage for the various positions of the flat target, which includes coefficients within 95% confidence intervals, an R-squared value of 0.9999, and a root square mean error (RSME) of 0.04121.

(5) $$V=-3060\times D^{-1.015}+11.26$$

From Equation (5), the distance (*D*) for the measured voltage (*V*) has been obtained:

(6) $$D=-{\left(\frac{1}{3060}\left(V-11.26\right)\right)}^{-\left(\frac{1}{1.015}\right)}$$

### 3.2. Experiment to Estimate Roughness Using 5.6 mm Capacitance Sensor

**Figure 8:**
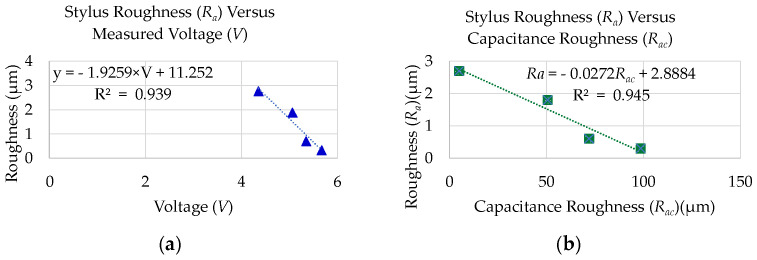
Roughness prediction model using experiment for selected specimen: (**a**) regression model for stylus roughness (*R_a_*) versus voltage (*V*); (**b**) regression model for stylus roughness (*R_a_*) Versus capacitance roughness (*R_ac_*).

Table 3 shows the mean distance computed for the measurement carried out with the stand-off distance (SOD) of 400 µm. Removing the SOD value from the mean distance (*Z_m_*) gives roughness measured through the capacitance sensor (*R_ac_*). *R_ac_* computed in Table 3 is only for selected rough surfaces. The linear regression model has been obtained by plotting the stylus-measured *R_a_* against the voltage (*V*) which has been shown in Equation (7) with an R^2^ value of 0.9379. This equation has been mentioned as capacitance roughness (*R_c_*_3_) used to calculate roughness from the obtained voltage. We used specimens with roughnesses of 0.3 µm, 0.6 µm, 1.8 µm, and 2.7 µm to create the regression model. This has been shown in Figure 8a. In addition, the linear regression model has been obtained by plotting the stylus-measured *R_a_* with the SOD-removed capacitance roughness (*R_ac_*) which has been shown in Equation (8) with an R^2^ value of 0.9450. This has been shown in Figure 8b and the equation has been mentioned as capacitance roughness (*R_c_*_4_) used to calculate roughness from the obtained SOD-removed distance. Both these equations are used to estimate the roughness of the remaining machined specimen. However, the samples with higher roughness (7.2 µm and 9.4 µm) values have not been considered because of the change in the pattern of the voltage for roughness change.

(7) $$R_{c3}=R_{a}=-1.9259\times V+11.252\;$$

(8) $$R_{c4}=R_{a}=-0.0272\times R_{ac}+2.8884\;$$

**Figure 9:**
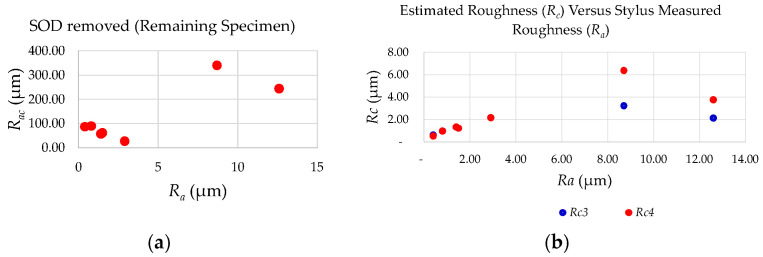
Response of estimated roughness for remaining specimens: (**a**) SOD removed from computed *Z_m_*; (**b**) estimated capacitance roughness (*R_c_*).

After estimating the capacitance roughness values (*R_c_*_3_ and *R_c_*_4_) of the remaining specimen by substituting the measured voltage (*V*) and SOD-removed capacitance roughness (*R_ac_*) in the regression model shown in Equations (7) and (8), respectively, the relative error has been calculated using Equation (9). The estimated capacitance roughness values, *R_c_*_3_ and *R_c_*_4_, have been listed in Table 4 for the remaining specimens. For higher roughness values, the deviation is large, and this has been plotted in Figure 9. Figure 9a shows that as the roughness (*R_a_*) increases, the *R_ac_* decreases, but the deviation is large for higher-roughness specimens. The estimated capacitance roughness (*R_c_*) for the remaining specimen is shown in Figure 9b. However, the SOD-removed *R_ac_* value has a larger deviation when compared to *R_a_*; this may be because of tilt and waviness in the specimen. To analyze this, finite element modeling and analysis were carried out, as detailed in Section 3.3. 

**Figure 10:**
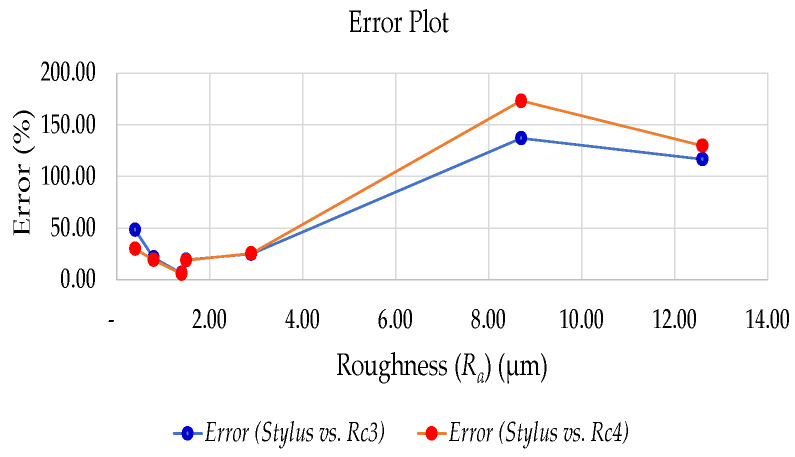
Error plot for the estimated *R_c_*_3_ and *R_c_*_4_ against measured *R_a_*.

The response of the C1-A capacitance sensor has shown a decreasing trend in voltage with an increase in roughness (*R_a_*) for the remaining specimens as well. From 0.4 µm to 2.9 µm, the voltage was decreasing. Above 2.9 µm, the deviation is higher, which means the sensor senses a higher gap between the sensing electrode and the target surface. This is due to the less capacitance sensed by the driver circuit, since the sensing area has very few peaks with maximum height. Figure 10 shows the error percentage plot for the value obtained from the roughness estimation equations *R_c_*_3_ and *R_c_*_4_ using the percentage error equation shown in Equation (9).

(9) $$\mathrm{Percentage}\;\mathrm{Error}\;=\left|\frac{Measured\;Value-Expected\;Value}{Expected\;Value}\right|\times 100$$

### 3.3. Model Development and Simulation Steps to Study Response of 5.6 mm Sensing Diameter Sensor for Flat and Rough Specimens using Finite Element Analysis

**Figure 11:**
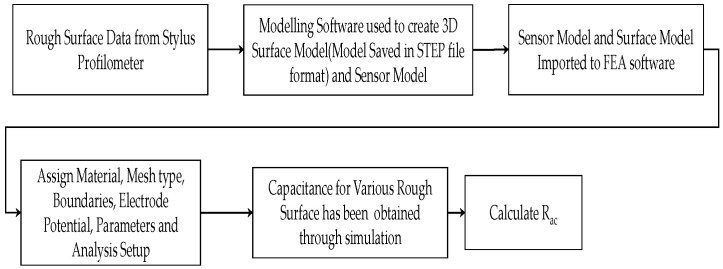
Simulation steps involved in capacitance sensor with surface (flat and rough) model.

An FEA simulation has been carried out for a selected specimen to understand and validate the sensor usage for roughness measurement. The diameter of the sensing electrode for capacitive sensors is considered as 5.6 mm. The dielectric material has been selected as epoxy between the sensing electrode and the inner guard ring to improve the homogeneity of electricity, as well as between the guard ring and the sensor inner guard. According to Maxwell [32], capacitance increases owing to field fringing into an annulus that is just a few parts per million larger in capacitance compared to the space between the sensor electrodes and the guard ring. Based on the simulation work carried out by Smith et al. [33,34] using the C1-C capacitance sensor, the material and potential level have been chosen. The dielectric width assumed has been well supported by assumptions by Maxwell. The steps followed to model the sensor and machined surface are illustrated in Figure 11. Air has been assigned as the medium between the sensor and the target surface. The inner guard, sensor body, and target material have been assigned as steel.

**Figure 12:**
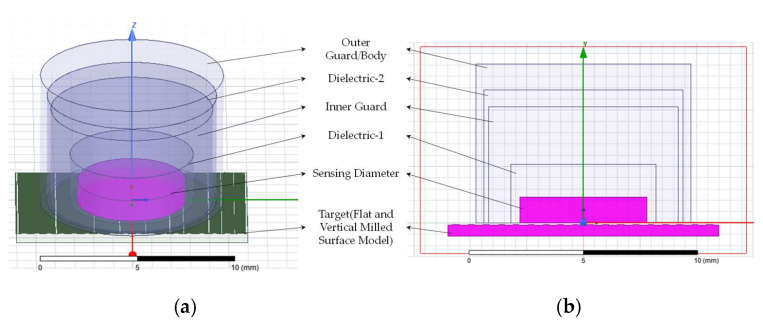

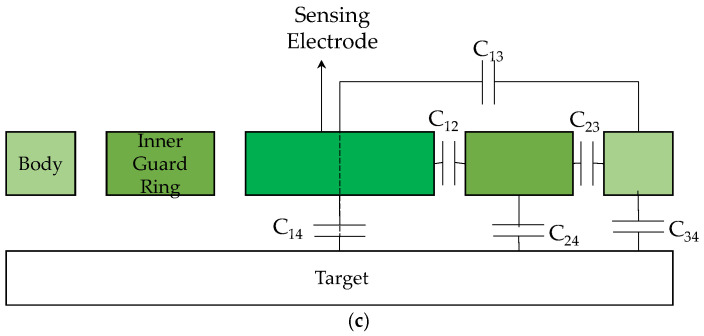
Sensor model: (**a**) 3D model of 5.6 mm capacitance sensor model with surface model; (**b**) 2D model of 5.6 mm capacitance sensor model with surface model (**c**); lumped capacitance between electrodes.

The 3D and 2D sensor models with rough surfaces are shown in Figure 12a,b, respectively. The potential difference has been assigned as 5 *V* for the sensing electrode and 0 *V* for the target surface. The inner guard and body have been assigned as 5 *V* and 0 *V*, respectively. To determine the relation between capacitance and distance changes for a flat surface target, the model has been moved from a minimum stand-off distance of 250 microns to 2250 microns following the calibration performed on a real sensor. Equation (10) gives the relation between the distance moved (D) and the corresponding capacitance change (C_14_). This relation has been obtained from the values listed in Table 2. Each tetrahedral element comprises 10 nodes. The meshing procedure was executed with the default parameters. Solution setup was allocated with a maximum of 10 passes and a 1% error rate. Capacitance matrix (CMATRIX) was computed using the FEA software (Ansys Maxwell 17, ANSYS, USA) to determine the energy stored between the electrodes. Figure 12c depicts the capacitance C_14_ between the sensor and the target. The simulation has been repeated with the same setting by fixing the stand-off distance of 500 µm between the sensor model and the vertically milled specimen model prepared from stylus profile data. The stylus profile data are waviness-removed and tilt-removed data which have the roughness information.

(10) $$D=Z_{m}=246.36\times C_{14}^{-1.009}$$

### 3.4. FEA Simulation Results for Vertically Milled Surface using 5.6 mm Capacitance Sensor Model 

The simulation has been performed using a sensor model and a surface model developed by following the steps presented in Figure 11. Table 5 shows the mean distance computed for the simulation carried out with the stand-off distance (SOD) of 500 µm. The removal of the SOD value from the mean distance (*Z_m_*) gives roughness measured through the capacitance sensor (*R_ac_*). *R_ac_* computed in Table 5 is only for a selected rough surface model. 

The FEA simulation shows linear variation for all roughness ranges. As mentioned before, the profile data used for developing the surface model have tilt and waviness removed, so the change in capacitance is purely due to roughness change. In the simulation, the sensor model shows good sensitivity and resolution; it shows considerable variation in roughness for a low to high range of roughness and can sense small changes in mean distance change (*Z_m_*). In addition, the removal of SOD from *Z_m_* directly gives the roughness value. However, in experiments using real sensors, there were many deviations due to error sources such as tilt and waviness. However, the simulation result also indicates that the 5.6 mm sensing diameter output has a better working range of 0.3 µm to 2.7 µm, above which there was variation, but the deviation was alarmingly large. 

### 3.5. Sensor Sensing Diameter Selection for Roughness Estimation 

As predicted, the sensor works better in the range of 0.3 µm to 2.9 µm, for which the 2D evaluation length mentioned in ISO4288/4287 is closer to the sensor diameter. In addition, before the selection of the sensor diameter, the FEA study results show that the sensor with 0.5 mm sensing diameter gives a considerable increase in capacitance for lower roughness, and for higher roughness, the response does not increase as happened in the lower roughness range. This has been reported in our previous work [27]. In this work, the 5.6 mm capacitance sensor works satisfactorily as predicted in the selected roughness range. This result shows strong evidence that the sensor sensing diameter needs to be selected based on the roughness range.

### 3.6. Stand-Off Distance Selection for Roughness Estimation

As discussed in Section 3.2, before measurement, the capacitance sensor was calibrated using an optical flat target, and the values for different positions are given in Table 2. The relation between the distance and the voltage has been obtained through this experiment. As mentioned in the earlier discussion, the simulation carried out with the 5.6 mm sensing diameter capacitance sensor model with a flat surface in the same distance as followed in the real sensor calibration step shows the same response as the real sensor. The real sensor calibration response chart and the simulation result with output C_14_ lumped capacitance have proportional changes. From this, it has been observed that the C1-A capacitance sensor uses a driver circuit that gives output voltage inversely proportional to the capacitance change. It is clear from the response chart in Figure 9 that a for stand-off distance of up to 500 µm, the sensor has higher sensitivity, and after 500 µm, the response to distance change shows a very minimal change.

## 4. Conclusions

In the present study, an endeavor was made to develop a capacitance-sensor-based micro gantry system using a single-shot approach for surface roughness measurement of vertically milled specimens. From the detailed analysis, it has been concluded that the proposed model can measure the surface roughness in the proposed approach.

- The developed gantry system approach can reduce error and setting time for achieving the required SOD before measurement.
- The capacitance sensor with a sensing diameter of 5.6 mm can perform well in the range of 0.3 µm to 2.9 µm roughness owing to the resolution constraints and area of measurement involved.
- FEA analysis results show that tilt and waviness are the error source in roughness measurement.
- Further, this method can be recommended for online measurement with the same machining conditions in mass production after considering suitable retrofitting arrangements for removing coolant during machining.

## Figures and Tables

**Table 1:** Specification of roughness measurement setup using capacitance sensor.

**Capacitive Sensor (Lion Precision C1-A)**	
Sensing Diameter	5.6 mm
Sensor Resolution (RMS)	478.92 nm
Near Gap	250 µm
Range	2000 µm
Output	0–10 VDC
Bandwidth	15,000 Hz
**XYZ Stage (PI—Physik Instrumente)**	
Travel Range	300 mm
Velocity	2.5 µm/s
Operating Voltage	12 V
Minimum Incremental Movement	0.2 µm
Resolution	0.018 µm

**Table 2:** Calibration of C1-A capacitance sensor.

Specimen No.	Distance to Target (D) (µm)	Capacitance Value (C_14_) (pF)	Real Sensor Output Voltage (*V*)
1	250.004	0.980	0.0
2	375.001	0.663	3.729
3	499.99	0.500	5.644
4	750.007	0.329	7.578
5	999.996	0.245	8.536
6	1250.004	0.201	9.094
7	1499.996	0.180	9.451
8	1749.997	0.153	9.694
9	2000	0.133	9.865
10	2250	0.118	9.992

**Table 3:** Experiment on calibration of sensor against selected specimens to obtain estimation model.

Specimen No.	Stylus Roughness *R_a_* (µm)	Measured Voltage (Volt)	Mean Distance (*Z_m_*) (µm)	Stand-Off Distance (SOD) Removed-(*R_ac_*) (µm)
1	0.3	5.668	498.495	98.495
2	0.6	5.348	471.959	71.959
3	1.8	5.063	450.540	50.540
4	2.7	4.354	404.910	4.910
5	7.2	7.516	740.222	340.222
6	9.4	6.949	644.224	244.224

**Table 4:** Estimation of roughness for the remaining machined specimens using regression model.

Specimen No.	SS Roughness *R_a_* (µm)	Measured Voltage (Volt)	Estimated Roughness (*R_c_*_3_) (µm)	Mean Distance (*Z_m_*) (µm)	Stand-Off Distance Removed (*R_ac_*) (µm)	Estimated Roughness (*R_c_*_4_) (µm)
7	0.4	5.534	0.59	487.035	87.035	0.52
8	0.8	5.338	0.97	489.867	89.867	0.95
9	1.4	5.163	1.31	457.819	57.819	1.32
10	1.5	5.213	1.21	461.549	61.549	1.21
11	2.9	4.716	2.17	426.993	26.993	2.15
12	8.7	7.516	3.22	740.222	340.222	6.37
13	12.6	6.949	2.13	644.224	244.224	3.75

**Table 5:** Stylus- vs. capacitance sensor (5.6 mm sensing diameter)-measured roughness (single-shot measurement)—software simulation.

Specimen No.	Stylus Roughness *R_a_* (µm)	Capacitance Value (C_14_) (pF)	Mean Distance (*Z_m_*) (µm)	Stand-Off Distance Removed (*R_ac_*) (µm)
1	0.3	0.4956	500.296	0.296
2	0.6	0.4965	499.371	0.629
3	1.8	0.4978	498.065	1.935
4	2.7	0.4983	497.480	2.520
5	7.2	0.5067	489.179	10.821
6	9.4	0.5140	482.179	17.821

## Data Availability

This study does not report any data.
